# Urinary metabolites associate with the rate of kidney function decline in patients with autosomal dominant polycystic kidney disease

**DOI:** 10.1371/journal.pone.0233213

**Published:** 2020-05-22

**Authors:** Shosha E. I. Dekker, Aswin Verhoeven, Darius Soonawala, Dorien J. M. Peters, Johan W. de Fijter, Oleg A. Mayboroda, on behalf of the DIPAK Consortium

**Affiliations:** 1 Department of Nephrology, Leiden University Medical Center, Leiden, the Netherlands; 2 Center for Proteomics and Metabolomics, Leiden University Medical Center, Leiden, the Netherlands; 3 Department of Internal Medicine, Haga Teaching Hospital, The Hague, The Netherlands; 4 Department of Human Genetics, Leiden University Medical Center, Leiden, the Netherlands; International University of Health and Welfare, School of Medicine, JAPAN

## Abstract

**Background:**

The variable course of autosomal dominant polycystic kidney disease (ADPKD), and the advent of renoprotective treatment require early risk stratification. We applied urinary metabolomics to explore differences associated with estimated glomerular filtration rate (eGFR; CKD-EPI equation) and future eGFR decline.

**Methods:**

Targeted, quantitative metabolic profiling (^1^H NMR-spectroscopy) was performed on baseline spot urine samples obtained from 501 patients with ADPKD. The discovery cohort consisted of 338 patients (56% female, median values for age 46 [IQR 38 to 52] years, eGFR 62 [IQR 45 to 85] ml/min/1.73m^2^, follow-up time 2.5 [range 1 to 3] years, and annual eGFR slope –3.3 [IQR –5.3 to –1.3] ml/min/1.73m^2^/year). An independent cohort (n = 163) was used for validation. Multivariate modelling and linear regression were used to analyze the associations between urinary metabolites and eGFR, and eGFR decline over time.

**Results:**

Twenty-nine known urinary metabolites were quantified from the spectra using a semi-automatic quantification routine. The model optimization routine resulted in four metabolites that most strongly associated with actual eGFR in the discovery cohort (F = 128.9, P = 7×10^−54^, R^2^ = 0.724). A model using the ratio of two other metabolites, urinary alanine/citrate, showed the best association with future annual change in eGFR (F = 51.07, P = 7.26×10^−12^, R^2^ = 0.150). This association remained significant after adjustment for clinical risk markers including height-adjusted total kidney volume (htTKV). Results were confirmed in the validation cohort.

**Conclusions:**

Quantitative NMR profiling identified urinary metabolic markers that associated with actual eGFR and future rate of eGFR decline. The urinary alanine/citrate ratio showed additional value beyond conventional risk markers.

## Introduction

Autosomal dominant polycystic kidney disease (ADPKD) is the most common hereditary renal disease with a prevalence of about 4:10.000, accounting for approximately 10% of patients on renal replacement therapy [1, 2]. The disease is caused by mutations in the *PKD1* or *PKD2* genes which accounts for 85% and 15% of cases, respectively. Progressive growth of cysts results in loss of functioning nephrons and progressive deterioration of renal function with approximately 50% of patients reaching end-stage renal failure by the age of 60 years. Patients and physicians need to know the prognosis regarding renal function to allow for family and career planning, and to decide who is eligible for renoprotective treatment and for participation in trials of novel therapies [3, 4]. Since the disease course of ADPKD is highly variable, it is important to identify the patients at high-risk of rapid disease progression. Conventional markers to predict disease progression, such as height-adjusted total kidney volume (htTKV) and a genotype assessment are time consuming and expensive. Several alternative serum and urinary biomarkers including neutrophil gelatinase-associated lipocalin, monocyte chemoattractant protein-1, beta*-*2 microglobulin, and soluble urokinase plasminogen activator receptor have been reported to have (at best) moderate predictive value [5, 6]. None of these markers are used in clinical care. Therefore, identification of new markers which reliably and robustly associate with disease severity and progression is relevant to patients with ADPKD.

Metabolomics is a post-genomic discipline, which offers an analytical framework for the discovery of novel diagnostic and predictive markers and may also provide insight into pathophysiological mechanisms in the study of renal diseases [7–9]. Metabolites, the intermediates and end-products of metabolism, measured in body fluids represent the physiological phenotype of an organism and its dynamic response to environmental influences, pathophysiological stimuli as well as biochemical adaptations of the biological systems [10, 11]. Nuclear magnetic resonance (NMR) spectroscopy and mass spectrometry are the main analytical technologies utilized in metabolomic strategies [7]. Compared to mass spectrometry, NMR may not appear as a very sensitive method, but this methodology offers unsurpassed analytical reproducibility, which often is a more valuable trait in clinical research [10, 12].

Analysis of the urine metabolome has been widely applied in chronic renal diseases [8], but data in patients with ADPKD are scarce. Urinary metabolic profiling in experimental models of polycystic kidney disease (PKD) have indicated that several metabolic pathways related to energy metabolism and cell proliferation were altered in affected animals [13–16]. Only one small cross-sectional study has investigated urinary metabolic profiles in patients with ADPKD. Urinary metabolic profiling by NMR differentiated ADPKD patients with well-preserved renal function from those on hemodialysis as well as type-2 diabetics with chronic kidney disease and healthy volunteers [17]. The alterations in NMR profiles or specific metabolites were, however, not quantified and not studied in relation to disease progression. We applied a quantitative NMR-based metabolomics strategy for urinary metabolic profiling in a large cohort of patients with ADPKD. The aim was twofold; first to explore changes in the urinary metabolome that associate with the actual eGFR and second to explore which baseline urinary metabolites associate with future progressive renal disease as assessed by annual eGFR decline over time. This is a first step towards identifying urinary markers for early risk stratification.

## Material and methods

### Study population and endpoints

#### Discovery cohort

This cohort consisted of ADPKD patients (n = 382) participating in the national DIPAK consortium (DIPAK1 trial: age 18–60 years, eGFR 30–60 ml/min/1.73m^2^, included from 2012–2015, n = 137; DIPAK observational study: age ≥18 years, eGFR ≥15 ml/min/1.73m^2^, included from 2013–2015, n = 189), and the Dutch Parelsnoer Institute/CuraRata biobank initiative (2010, n = 56). All samples from the DIPAK 1 trial were collected at baseline before lanreotide treatment [18]. The first 137 patients enrolled in the DIPAK 1 trial were included in the discovery phase in addition to patients from the two observational studies. *Validation cohort*: The ADPKD patients that were subsequently enrolled in the DIPAK 1 trial (n = 172) were included as validation cohort in the second phase of our study. None of the patients used tolvaptan. The use of other concomitant treatment was not restricted in any of the cohorts.

The diagnosis ADPKD was based upon the modified Ravine criteria [19] and/or genetic mutation analysis. The DIPAK 1 trial (ClinicalTrials.gov identifier: NCT01616927) and the DIPAK observational study were centrally approved by the Medical Ethics Committee of the University Medical Center Groningen, and additionally by the institutional review boards of all study centers participating in the national DIPAK consortium (Leiden University Medical Center, Leiden; Radboud University Medical Center, Nijmegen; Erasmus Medical Center, Rotterdam). Analysis of the cohort of the Dutch Parelsnoer Institute/CuraRata initiative did not require additional ethics committee board approval since these samples were obtained from a biobank. Subjects from all studies provided written informed consent. Consent included storage of data and samples and use of samples in future biomedical research such as ours. Patients provided written informed consent to have data from their medical records used in research. All data/samples in the clinical databases and biobank were fully anonymized before we accessed them.

We analyzed the association between metabolic profiles in baseline urine samples and actual eGFR using the CKD-EPI equation [20]. Furthermore, we analyzed the association between metabolic profiles in these samples and the subsequent annual change in eGFR over time. In the DIPAK 1 trial, eGFR measurements were performed at baseline, week 12, and every 12 weeks thereafter until week 132, whereas in the observational study and Parelsnoer biobank initiative, annual eGFR measurements were available. The annual change in eGFR during follow-up was calculated using linear regression slopes through serial eGFR measurements and was expressed as the change in ml per year. Patients with a follow-up time <1 year were excluded from the eGFR slope analyses.

### Urine samples collection and preparation

Standard methods were used to collect, process and store the early morning void urine samples. These urine samples were collected under overnight-fasting conditions. They were collected in sterile containers and centrifuged at 1000g for 10 minutes. The supernatant was processed into aliquots of 2ml and stored at -80°C until analysis. Sampling of fresh urine to measure pH was not part of the protocol. All samples were stored for two to eight years and underwent one to two freeze-thaw cycles. For sample preparation, samples were thawed, transferred into 96 deep-well plates and centrifuged at 1550 g for 5 minutes. Using a Gilson 215 liquid handler, 630μl of urine was mixed with 70μl of pH 7.4 phosphate buffer (1.5 M) in 100% D_2_O containing 4mM TSP and 2mM NaN_3_. A customized Gilson 215 liquid handler was used to transfer the samples to a 5.0mm Bruker NMR tube rack.

### NMR spectroscopy

^1^H NMR data were collected using a Bruker 600MHz AVANCE II spectrometer equipped with a 5mm TCI cryogenic probe head and a z-gradient system. A Bruker SampleJet was used for sample insertion and removal. All experiments were recorded at 300K. A fresh sample of 99.8% methanol-d4 was used for temperature calibration [21] before each batch of measurements. Duration of 90° pulses were automatically calibrated for each individual sample using a homonuclear-gated mutation experiment [22] on the locked and shimmed samples after automatic tuning and matching of the probe head. One-dimensional (1D) ^1^H NMR spectra were recorded using the first increment of a NOESY pulse sequence [23] with presaturation (γB_1_ = 50Hz) during a relaxation delay of 4s and a mixing time of 10ms for efficient water suppression [24]. Initial shimming was performed using the TopShim (Bruker Corporation, 2011) tool on a random mix of urine samples from the study, and subsequently the axial shims were optimized automatically before every measurement. Sixteen scans of 65,536 points covering 12,335Hz were recorded. J-resolved (JRES) spectra were recorded with a relaxation delay of 2s and two scans for each increment in the indirect dimension. A data matrix of 40×12,288 data points was collected covering a sweep width of 78×10,000 Hz.

### Identification and quantification of metabolites

Peaks were assigned to specific metabolites using a variety of techniques. Abundant metabolites were identified using the list presented in the human urine metabolome database and by comparing the chemical shift with the one listed in the HMDB [25], BMRB [26] or Chenomx software (Chenomx Inc., 4232–10230 Jasper Ave, Edmonton, Alberta, Canada). Different peaks that originate from the same metabolite have a perfect correlation, which also helps with peak identification. TOCSY, edited HSQC and HMBC two-dimensional (2D) NMR spectra of a mixed sample provided additional information for metabolite quantification. When the techniques above were not sufficient to narrow the peak assignment down to a single metabolite, spiking of pure compound into a urine sample was used to determine which metabolite matched the peak. Metabolomic analysis of body fluids offers two strategies: untargeted where all acquired data is used for the analysis, and the targeted where the analysis is based on a subset of the annotated structures. The first approach appears to be an attractive discovery tool, but its practical value is often strongly reduced by difficulties during the annotation of the discovered spectral regions. The targeted approach limits the analysis to a predefined set of structures, but simplifies the interpretation and increases the translational value of findings. The urinary metabolic profiles of patients were extracted using a targeted semi-automatic NMR quantification workflow (KIMBLE) [27]. The selection of metabolites that were quantified was data driven and based on the well-defined HMDB list of urinary metabolites [12]. In total, 31 urinary metabolites were identified and quantified. Leaving out ethanol because of its dietary origin and fumarate because of its relatively low quantification quality, analyses were performed with 29 metabolites. To compensate for urine dilution differences, the data were normalized using probabilistic quotient normalization (PQN), a normalization routine specifically developed for complex NMR data. The data is scaled on the basis of the most probable dilution, which is estimated from the analysis of the reference spectra [28].

### Statistical analysis

The data analysis and visualization were performed with R versions 3.51 and 3.2.3, and Python version 2.7.12. Regarding the descriptive statistics, non-normally distributed data were expressed as median with interquartile range (IQR). Categorical data were given as proportions. Differences between variables were tested using a Mann-Whitney *U* test when not normally distributed. A chi-square test was used in case of categorical data. Data was not corrected for treatment with lanreotide, since all samples were collected at baseline before lanreotide was started, and lanreotide did not affect the rate of eGFR decline over time [29]. Multivariate linear regression analyses were used to explore associations between urinary metabolites and eGFR progression. Parameters which were associated with annual eGFR slope in univariate regression analyses (age, sex, baseline eGFR and baseline htTKV) were included as independent variables in the multifactorial model, resulting in exclusion of albuminuria. The following metrics were reported for these models: standardized β coefficients (showing how many SD a dependent variable will change, per SD increase in the predictor variable), the result of the F-test of the overall significance (describing a difference between a model with no predictors and the tested model), its P-value, and R^2^ (showing the percentage of the variance in the dependent variable that the independent variables explain). For the linear modeling and linear model diagnostics, the basic *lm* function, the *caret* package and the *car* package were used. Model optimization and variable selection was performed using the best subset selection approach, an exploratory model building regression analysis which compares all possible models that can be created based upon an available set of the variables. The *regsubset* function of the *leaps* package was used for the selection. It tested all possible combinations of the metabolites and all their respective ratios to find the best-performing linear model. A combination of four variables was sought as a good compromise between model simplicity and performance. All visualizations were made with help of the *ggplot2* package, the predictor effect plot was generated using *effects* package [30], while the correlation heatmap was generated by the *corrplot* package.

## Results

### Discovery cohort

#### Patient characteristics

After NMR quality control of the spot urine samples, 338 patients were included in the discovery cohort. The characteristics of this cohort are summarized in Table 1. Patients had a median age of 46 (IQR 38–52) years and median eGFR of 62 (IQR 45–85) ml/min/1.73m^2^. Height-adjusted total kidney volume (htTKV) was available for 224 patients (median 935 [IQR 575–1398] ml/m). Out of the 338 patients, 309 patients had a follow-up of ≥1 year, and were included in the eGFR slope analysis (median age 46 [IQR 39–52] years, median eGFR 62 [IQR 45–85] ml/min per 1.73m^2^, median follow-up time of 2.5 [range 1–3] years, and median annual change in eGFR -3.3 [IQR -5.3 –-1.3] ml/min/1.73m^2^).

**Table 1:** Baseline characteristics of ADPKD patients in the discovery and validation cohort.

Variable	Discovery cohort	Validation cohort	*P* value*
*n*	338	163	
Female sex, *n* (%)	190 (56)	87 (53)	0.55
Age, years	46 (38–52)	49 (44–55)	<0.001
Height, m	1.75 (1.68–1.84)	1.77 (1.70–1.84)	0.18
BMI, kg/m^2^	26 (23–28)	26 (24–29)	0.60
eGFR, ml/min per 1.73m^2^	62 (45–85)	50 (41–58)	<0.001
HtTKV, ml/m	935 (575–1398)	1142 (775–1778)	0.001
Urine ACR, mg/mmol	2.8 (1.1–6.4)	4.2 (2.3–9.6)	<0.001
Gene type mutation, *n*	287	158	
*PKD1*, *n* (%)	222 (77)	116 (73)	0.06
*PKD2*, *n* (%)	50 (18)	39 (25)	
None, *n* (%)	15 (5)	3 (2)	

Data in median and interquartile ranges. ACR, albumin-to-creatinine ratio; ADPKD, autosomal dominant polycystic kidney disease; eGFR, estimated glomerular filtration rate; htTKV, height-adjusted total kidney volume.

*P values were calculated using a Mann-Whitney *U* test in case of non-normally distributed data, and a chi-square test in case of categorical data.

Twenty-nine urinary metabolites were quantified using a targeted semi-automatic NMR quantification workflow and were used for analyses. Probabilistic quotient normalization (PQN) was used to correct for differences in urine dilution. PQN values showed a strong correlation with both urinary osmolality (*r* = 0.86, S1A Fig) and urinary creatinine (*r* = 0.84, S1B Fig). Concentrations of all quantified metabolites within the different CKD stages are reported in S1 Table. The principal component analysis model showed that urinary metabolic profiles did not differ between urine samples obtained under fasting or non-fasting conditions (S2 Fig).

#### Association of urine metabolites with baseline eGFR

**Fig 1:**
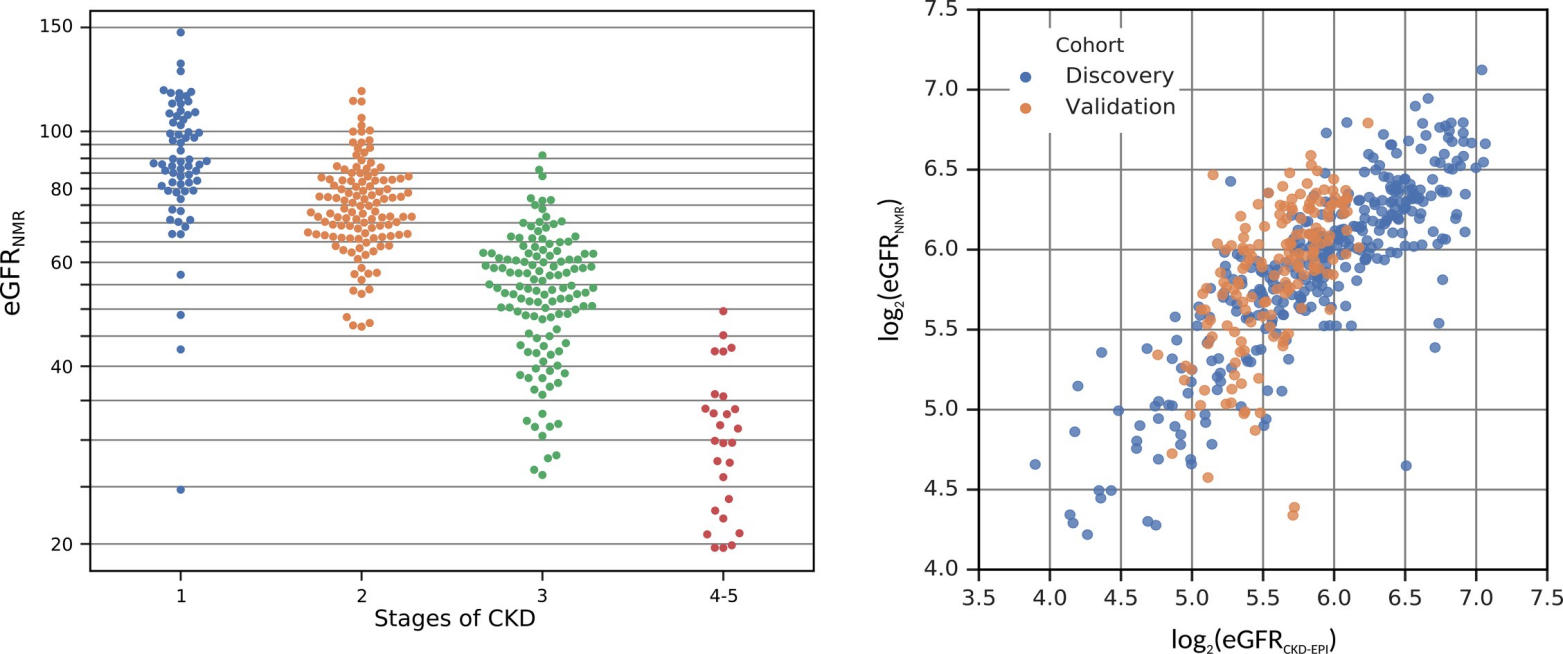
**A.** Swarm plot showing a distribution of the predicted estimated glomerular filtration rate (eGFR) versus the different stages of chronic kidney disease (CKD). The nuclear magnetic resonance (NMR)-derived eGFR model for prediction based on four urinary metabolites (myo-inositol, asymmetric dimethylarginine (ADMA), 3-hydroxyisovalerate, creatinine) reliably explained the different stages of CKD. **B.** The model including four urinary metabolites associated with actual estimated glomerular filtration rate (eGFR). A linear model (transformed to log_2_ eGFR) based on four urinary metabolites (myo-inositol, asymmetric dimethylarginine (ADMA), 3-hydroxyisovalerate, creatinine) served as a good predictor for eGFR (F = 128.9, P = 7×10^−54^, R^2^ = 0.724) in the discovery cohort (blue dots), and could be validated in an independent cohort (orange dots). The Pearson correlations between measured and predicted eGFR values are *r* = 0.85 and *r* = 0.65 in the discovery and validation cohort, respectively).

The first step of our analysis was to evaluate whether urinary metabolites associate with renal function. The correlation map shows that several metabolites correlated with baseline eGFR (S3 Fig). To overcome potential issues of multiple regression such as multicollinearity which leads to a strong overestimation of the model quality, we applied a model optimization procedure. The aim of such an exploratory algorithm is to evaluate all possible combinations of the 29 quantified urinary metabolites (predictors) and find an optimal combination for prediction of the baseline eGFR. The automatic optimization procedure selects the statistically best combination of metabolites for the model, considering the interaction between metabolites. The most optimal model, therefore, does not necessarily include the individually most significantly associated metabolites (S1 Table). The optimal model that was obtained, was based on a subset of four urinary metabolites: myo-inositol, asymmetric dimethylarginine (ADMA), 3-hydroxyisovalerate and creatinine. This model served as a good predictor for eGFR (F = 128.9, P = 7×10^−54^, R^2^ = 0.724). Fig 1A represents a plot showing that the predicted eGFR values based on the model consisting of four urinary metabolites correctly defined the actual CKD stage. Fig 1B represents a plot of the calculated versus the predicted eGFR. It shows that the predicted eGFR values strongly associated with the calculated eGFR values (blue dots, *r* = 0.85).

#### Association of urine metabolites with eGFR slope

**Fig 2:**
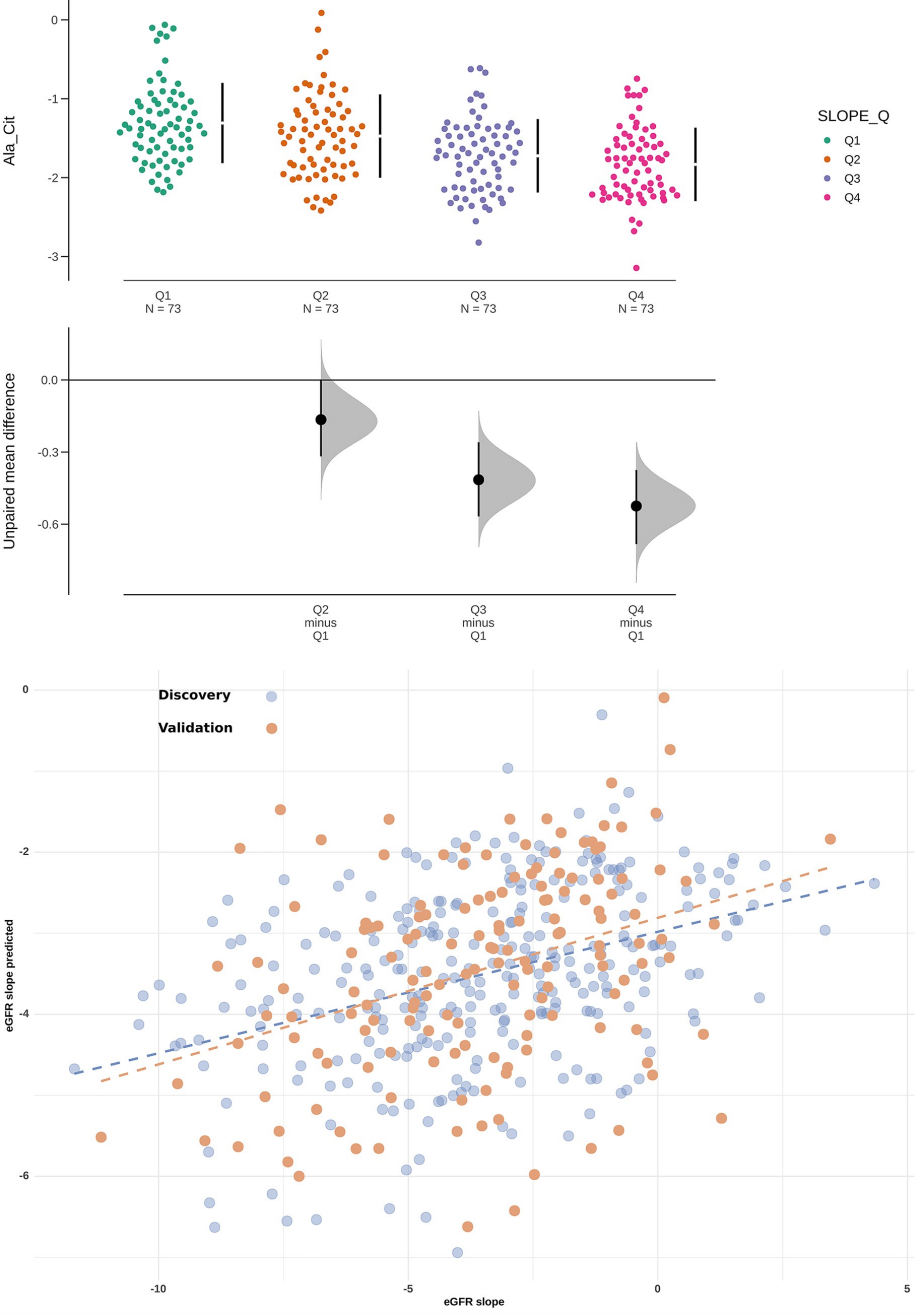
**A.** Swarm plot showing the differences between the urinary alanine/citrate ratio within quartiles of the annual eGFR slope. Patients with more rapidly progressive renal function decline (green dots) showed a higher urinary alanine/citrate ratio. Upper part of the figure: mean±SD; lower part of the figure: the effect size and 95% CI's. Quartiles of annual change in eGFR (ml/min/1.73m^2^), expressed in slope ranges: Q1 = [-11.7, -5.14), Q2 = [-5.14, -3.28), Q3 = [-3.28, -1.41), Q4 = [-1.41,4.34]. **B.** The model including the urinary alanine/citrate ratio associated with annual change in estimated glomerular filtration rate (eGFR). The values for eGFR slope based on a model including the urinary alanine/citrate ratio associated with the measured annual change in eGFR (F = 51.07, P = 7.26×10^−12^, R^2^ = 0.150) in the discovery cohort (blue dots), and could be validated in an independent cohort (orange dots). The Pearson correlations between the measured and predicted eGFR slope values are *r* = 0.39 and *r* = 0.35 for the discovery and validation cohort, respectively).

To predict disease progression, we evaluated whether baseline concentrations of the 29 urinary metabolites associate with the future rate of annual decline in eGFR. We used the annual change in eGFR as a response variable and the quantified metabolites plus all their binary ratios as the predictors (449 features in total). Using the same model optimization routine as for baseline eGFR, we found that the alanine/citrate ratio was most strongly associated with the future annual change in eGFR. Patients with a more rapid decline in renal function had a higher baseline alanine/citrate ratio as compared with those with a slower decline in eGFR (Fig 2A). Fig 2B shows that the calculated annual eGFR slope associated with the eGFR slope that was predicted based on the alanine/citrate ratio. Tables 2 and 3 summarize the statistical metrics of the different regression models with and without the alanine/citrate ratio, showing that the models with the ratio remained significant after correction for clinical characteristics that are conventionally used for risk prediction (sex, age, and/or baseline eGFR, and/or htTKV). These tables also include the metrics of the models built only on the conventional clinical risk markers including baseline eGFR (Table 2) and baseline htTKV (Table 3). The model built on the alanine/citrate ratio is the most optimal one (F = 51.07, P = 7.26×10^−12^, R^2^ = 0.150). Also after adjustment for all conventional risk markers, it outperformed the model built only on age, sex and baseline eGFR (F = 7.31, P = 9.75×10^−5^, R^2^ = 0.071 versus including the alanine/citrate ratio: F = 14.61, P = 6.97×10^−11^, R^2^ = 0.169) or on baseline htTKV (F = 9.00, P = 1.03×10^−5^, R^2^ = 0.087 versus including the alanine/citrate ratio: F = 16.50, P = 3.66×10^−11^, R^2^ = 0.190). However, even the strongest model explains only a fraction of the variation in the data (about 15%). Analysis after random partitioning of a training cohort showed similar results for the performance of the model (S2 Table) as compared with the method that was applied in this study. We also repeated model derivation on a restricted cohort including only patients with at least two years of follow-up and/or four eGFR measurements for calculation of the eGFR slope. Using the same model optimization routine, the alanine/citrate ratio is still on top of the list candidate predictors, confirming that this ratio was most strongly association with annual eGFR decline (S4 Fig). A model with strongly significant predictors, but low R^2^ is evidently sub-optimal for prediction in clinical practice. Nonetheless, it shows a clear dependency between a given metabolic ratio and annual change in eGFR, which outperformed conventional clinical risk markers. Of note, using log_2_ transformed htTKV like in a previous study [31], the model including log_2_htTKV and the alanine/citrate ratio explained 21% of the variation in the data.

**Table 2:** Associations between annual change in eGFR and demographic variables and baseline eGFR with and without the alanine/citrate ratio.

Variable	St. β	F	*P*	R^2^
**Alanine/citrate ratio**	- 0.3869	51.07	7.26×10^−12^	0.150
+ age	-0.3858	26.90	1.93×10^−11^	0.157
	0.0851			
+ baseline eGFR	-0.3829	25.51	6.28×10^−11^	0.150
	0.0183			
+ age + baseline eGFR	-0.3598	18.94	3.09×10^−11^	0.165
	0.1533			
	0.1144			
Alanine/citrate ratioᵃ	- 0.3695	26.19	3.52×10^−11^	0.153
+ age	-0.3669	18.46	5.56×10^−11^	0.161
	0.0887			
+ baseline eGFR	-0.3658	17.44	1.98×10^−10^	0.154
	0.0172			
+ age + baseline eGFR	- 0.3400	14.62	6.97×10^−11^	0.169
	0.1580			
	0.1160			
**Baseline eGFR**	0.1022	3.06	0.081	0.010
+ age	0.2331	6.91	0.001	0.046
	0.2406			
Baseline eGFRᵃ	0.0899	5.29	0.005	0.035
+ age	0.2338	7.31	9.75×10^−5^	0.071
	0.2286			

^ᵃ^ model corrected for sex. eGFR, estimated GFR; st β, standardized β. St β, F and P values were calculated using multivariable linear regression. Dependent variable: annual change in eGFR, independent variables: alanine/citrate ratio, sex, age and baseline eGFR.

**Table 3:** Association between annual change in eGFR and demographic variables and baseline htTKV with and without the alanine/citrate ratio.

Variable	St. β	F	*P*	R^2^
**Alanine/citrate ratio**	- 0.3869	51.07	7.26×10^−12^	0.150
+ age	-0.3858	26.90	1.93×10^−11^	0.157
	0.0851			
+ baseline htTKV	-0.3477	33.26	1.00×10^−13^	0.187
	-0.1973			
+ age + baseline htTKV	-0.3412	21.98	7.99×10^−13^	0.189
	-0.1964			
	0.1054			
+ baseline log_2_(htTKV)/age˟	-0.3613	27.75	9.83×10^−12^	0.163
	-0.1414			
Alanine/citrate ratioᵃ	- 0.3695	26.19	3.52×10^−11^	0.153
+ age	-0.3669	18.46	5.56×10^−11^	0.161
	0.0887			
+ baseline htTKV	-0.3400	20.50	4.76×10^−12^	0.179
	-0.1826			
+ age + baseline htTKV	-0.3348	16.50	3.66×10^−12^	0.190
	0.1061			
	-0.1906			
**Baseline htTKV**	-0.2495	18.09	1.90×10^−5^	0.062
+ age	-0.2584	11.75	1.25×10^−5^	0.076
	0.1194			
+ baseline eGFR	-0.2481	9.43	0.0001	0.062
	0.0004			
Baseline htTKVᵃ	-0.2238	11.10	2.28×10^−5^	0.073
+ age	-0.2451	9.00	1.03×10^−5^	0.087
	0.1121			
Baseline log_2_(htTKV)/age˟	-0.1871	10.34	0.0014	0.035

^ᵃ^ model corrected for sex.

˟ model related to the Mayo classification. eGFR, estimated GFR; htTKV, height-adjusted total kidney volume; st β, standardized β. St β, F and P values were calculated using multivariable linear regression. Dependent variable: annual change in eGFR, independent variables: alanine/citrate ratio, sex, age and htTKV.

### Validation cohort

#### Patient characteristics

Of the patients that were selected for the independent validation phase, nine were excluded after NMR quality control, leaving 163 ADPKD patients (median age 49 [IQR 44–55] years, median eGFR 50 [IQR 41–58] ml/min/1.73m^2^, median htTKV 1142 [IQR 775–1778] ml/m) for analyses (Table 1). For 150 patients, a follow-up of at least one year was available. Median age was 49 [IQR 44–55] years, eGFR 50 [IQR 41–58] ml/min/1.73m^2^), follow-up time 2.5 (range 1.2–2.5) years, and annual change in eGFR -3.3 (IQR -5.3 –-1.6) ml/min/1.73m^2^. Patients in the validation cohort were relatively older and on average had a lower eGFR than the patients in the discovery cohort. For the discovery phase, we included a diverse cohort of ADPKD patients with a wide variety in age and stage of disease for identifying metabolites associated with eGFR. The independent cohort was included in our study to validate the results. Based on availability, this group consisted of a subset from the DIPAK 1 trial. The validation cohort is therefore characterized by a narrower distribution of baseline characteristics including age and renal function.

#### Association of urine metabolites with baseline eGFR

The model to predict baseline eGFR, based on the combination of four urinary metabolites, was validated in an independent cohort. Fig 1B shows that the calculated eGFR values strongly correlated with the predicted values in the validation cohort (orange dots; F = 31.10, P = 3.55×10^−19^, Pearson correlation coefficient *r* = 0.65), confirming the results found in the discovery cohort (*r* = 0.85; blue dots).

#### Association of urine metabolites with eGFR slope

The model including the urinary alanine/citrate ratio was validated in the independent cohort. Fig 2B shows that the calculated eGFR slope values correlated with the predicted eGFR slope in the validation cohort (orange dots; F = 20.71, P = 1.08×10^−5^, Pearson correlation coefficient *r* = 0.35), confirming the results found in the discovery cohort (*r* = 0.39; blue dots).

## Discussion

Our study provides the first systematic evaluation of the association between the urinary metabolome and severity and progression of disease in patients with ADPKD. Using targeted, quantitative ^1^H NMR based metabolic profiling in a large cohort of patients with different stages of CKD, we identified an optimal subset of four urinary metabolites (myo-inositol, 3-hydroxyisovalerate, ADMA and creatinine) that strongly associated with the actual eGFR. Furthermore, we showed that the ratio of baseline urinary alanine over citrate was the strongest combination of urinary metabolites associated with the subsequent rate of eGFR decline over time. These results were validated in a separate cohort of patients with ADPKD.

There is a need to identify biomarkers to predict the rate of eGFR decline, and to improve the current risk assessment strategies. Patients want to know their prognosis, and it is important to tailor treatments to individual patients since those at high-risk of rapidly progressing disease are most likely to benefit from treatment. Multiple studies have evaluated the use of biomarkers and clinical variables for predicting disease progression. The current gold-standard is a prognostic model based on htTKV and age (Mayo classification) [31]. In our study, the model including the urinary alanine/citrate ratio showed additional value beyond that of a model based on htTKV and age.

Data on urinary metabolic profiling in patients with ADPKD are still scarce. The only published report by Gronwald and colleagues described urinary profiles measured with ^1^H NMR in 54 patients with ADPKD with relatively preserved renal function, other patients with chronic kidney disease including 52 type 2 diabetics and 46 healthy volunteers. Their study focused on differences in the urinary metabolic profiles between the study groups. The association between the metabolic profile and ADPKD progression over time was not evaluated [17]. They also recorded 1D ^1^H and 2D ^1^H-^13^C HSQC NMR spectra for metabolite quantification. The 2D ^1^H-^13^C HSQC spectrum provides excellent resolution, but low signal-to-noise, and takes long to record. Our method is based on 1D NOESY and 2D JRES experiments, both purely ^1^H, which are quicker to record and allowed us to measure a greater number of samples. Because of the use of different NMR experiments, our selection of metabolites is not identical to the list provided by Gronwald et al. [17], although there is considerable overlap. Our report extends the limited pool of data on the urinary metabolome in ADPKD. Our study design is not suitable for an unequivocal causal or mechanistic interpretation of the findings. Understanding the functional and causal relationships between the set of predictors and renal function would require a different study design ideally in a large patient cohort with different stages of CKD, including patients with ADPKD. Having said that, the metabolites we discuss here in association with eGFR and eGFR loss have been mentioned in a context of a renal (patho)physiology more than once. The bulk of myo-inositol is produced by the kidneys [32]. In CKD, urinary levels of myo-inositol increase due to reduced tubular reabsorption [33]. ADMA is implicated with chronic kidney disease [34, 35]. An impaired renal glomerular filtration rate results in increased urine levels. Citrate is frequently mentioned as a “window to the renal metabolism” and its role in renal (dys)function is well documented [36]. Multiple studies have shown that citrate levels in plasma and urine decrease as CKD and ADPKD progress [17, 37–39]. Alanine, on the other hand, is not frequently mentioned in the context of renal physiology. A change in urinary alanine excretion following damage to the proximal tubule has been previously reported in a mercury-induced nephrotoxicity model [40]. Alanine aminopeptidase, a proximal tubule brush border enzyme, may be a marker of kidney damage. Interestingly, Gronwald et al. [17] reported reduced urinary levels of alanine in early stage ADPKD (mean eGFR 95.5±27.7 ml/min/1.73m^2^). In contrast, in the current study including predominately later-stage patients, we found increased levels, suggesting impaired reabsorption. Alternatively, the altered urinary alanine excretion in our study could be explained by a reduction in alanine aminopeptidase secreting cells due to progressive tubular damage. Based on the literature, we think that the metabolites that were identified play a role in chronic renal dysfunction in general, and may not be specific to ADPKD. A model including all quantified metabolites reliably distinguished ADPKD and other chronic, renal disease patients (CKD stage 1–2, mainly chronic glomerulonephritis) from healthy controls (data not shown). An association was found between the actual and predicted eGFR in both ADPKD (blue dots) and non-ADPKD (orange dots) patients (S5 Fig). Although this association was more scattered in non-ADPKD patients (between the cohorts; *r* = 0.289, P = 0.03), a definitive conclusion whether the identified model is ADPKD-specific could not be drawn because of the limited number of patients and eGFR range in the non-ADPKD cohort.

This study has a number of strengths. First, this is a large and phenotypically well-defined cohort of patients with ADPKD, including data on genetic mutation and total kidney volume for most participants. Second, most samples were collected and stored in a uniform way, limiting potential bias. Third, we used NMR as the methodology for the measurement of urinary metabolites, which is a robust and reproducible analytical method [10]. Our NMR work-flow has been specified and largely automated, making it possible to reliably and accurately measure and quantify a large group of urine metabolites in a large group of patients [27]. Finally, the results were confirmed in a validation cohort of patients with ADPKD.

This study also has limitations. The eGFR slopes were based on a median follow-up time of 2.5 years, which is relatively short compared with studies that were used for validation of htTKV to predict renal disease progression [31, 41]. This limited follow-up period may reduce the reliability of the eGFR slope to predict long-term disease progression and kidney failure. A shorter follow-up period may also make the eGFR slope more susceptible to random variance in eGFR at the time of sampling. Nevertheless, despite this, the association between the alanine/citrate ratio and eGFR slope could be validated in a separate cohort. Additionally, the validation cohort only consisted of patients with later-stage CKD. Future validation of our findings in patients with CKD stages 1–2 with a longer follow-up period is required to further establish the value of the selected ratio to predict the rate of renal function decline. Of note, in the discovery cohort, in a sub-selection of patients with CKD stages 1 and 2 (n = 185), the alanine/citrate ratio had a stronger association with annual change in eGFR (F = 29.06, P = 2.67×10^−7^, R^2^ = 0.16) than htTKV (F = 17.55, P = 4.80×10^−5^, R^2^ = 0.10). In this sub-analysis, a combined model with both the alanine/citrate ratio and htTKV performed best (F = 24.86, P = 5.11×10^−10^, R^2^ = 0.25).

In conclusion, this study shows the potential of urinary metabolic profiling in chronic kidney disease patients. Using quantitative NMR profiling we identified urinary metabolic markers that correlated with eGFR and with the future rate of decline in eGFR. Although our model with strongly significant predictors, but low R^2^ is evidently sub-optimal for prediction in clinical practice, it showed a clear dependency between the urinary alanine/citrate ratio and annual change in eGFR, which outperformed conventional clinical risk markers in early and late stage CKD. A study with a longer follow-up period and a larger cohort of patients with early stage ADPKD is needed to further explore the value of this ratio as a tool to predict disease progression.

## Supporting information

S1 Fig Correlation between probabilistic quotient normalization (PQN) and urinary osmolality (A) and urinary creatinine (B). To compensate for urine dilution differences, the metabolic data were normalized using PQN. This is a well-established normalization routine specifically developed for complex nuclear magnetic resonance spectroscopy (NMR), which considers the concentrations of all metabolites. The PQN scaling factor was correlated with (clinical laboratory-derived) urinary osmolality (A, *r* = 0.86). A similar correlation was found between PQN and urinary creatinine (B, *r* = 0.84).
(PDF)

S2 Fig A Principal Component Analysis (PCA) score plot comparing the urinary metabolic profiles between fasting and non-fasting obtained urine samples. The plot showed no trend associated with fasting status. It was built using data matrix of the 29 quantified metabolites, and the model required seven components to cover the first 50% of the variance with 20% covered by the first two components.
(PDF)

S3 Fig Correlation map of all quantified metabolites built on the discovery cohort. Several urinary metabolites correlated with the actual eGFR. Significant Pearson correlations (*r*, p<0.05) are indicated by circles, blue and red indicating positive and negative correlations, respectively. The size of the correlation is indicated by the shade of the circles, and is defined by the colour bar. Correlation clusters and the correlations of eGFR with the metabolites are highlighted by black and red borders, respectively.
(PDF)

S4 Fig Variable importance plot of a random forest regression model showing the top 10 of candidate predictors for estimated GFR (eGFR) progression. We performed model derivation based on a restricted cohort (n = 240) including patients with a follow-up of at least two years and/or four estimated GFR (eGFR) measurements to identify metabolites association with annual change in eGFR. The alanine/citrate ratio is most strongly associated with annual change in eGFR. Legend: x-axis; percentage increase in mean squared error (%IncMSE) when a variable is dropped, y-axis: metabolite ratios.
(PDF)

S5 Fig Correlation between actual and predicted estimated glomerular filtration rate (eGFR) in patients with Autosomal Dominant Polycystic Kidney Disease (ADPKD) and other chronic, renal disease patients (non-ADPKD). The model (transformed to log_2_ eGFR) including four urinary metabolites (myo-inositol, asymmetric dimethylarginine (ADMA), 3-hydroxyisovalerate, creatinine) was associated with the actual eGFR in ADPKD (n = 338, blue dots) and non-ADPKD (n = 42, CKD stage 1–2; orange dots) patients (between the cohorts; *r* = 0.289, P = 0.027).
(PDF)

S1 Table Probabilistic Quotient Normalization (PQN)-corrected levels of all quantified urinary metabolites (n = 29) stratified by CKD stage. Data in mean and SD. ADMA, asymmetric dimethylarginine; CKD, chronic kidney disease; DMA, dimethylamine; TMA, trimethylamine. *Between stages of CKD (Kruskal-Wallis test).
(PDF)

S2 Table Association between the urinary alanine/citrate ratio with annual change in eGFR in a randomly selected ADPKD cohort (n = 350). ADPKD, autosomal dominant polycystic kidney disease; eGFR, estimated GFR; st β, standardized β. St β, F and P values were calculated using multivariable linear regression.
(PDF)
