# Supplementary figures and images for: Urinary metabolites associate with the rate of kidney function decline in patients with autosomal dominant polycystic kidney disease
Source: PLoS One. 2020 May 22;15(5):e0233213. doi: 10.1371/journal.pone.0233213 (PMC7244119; doi:10.1371/journal.pone.0233213)

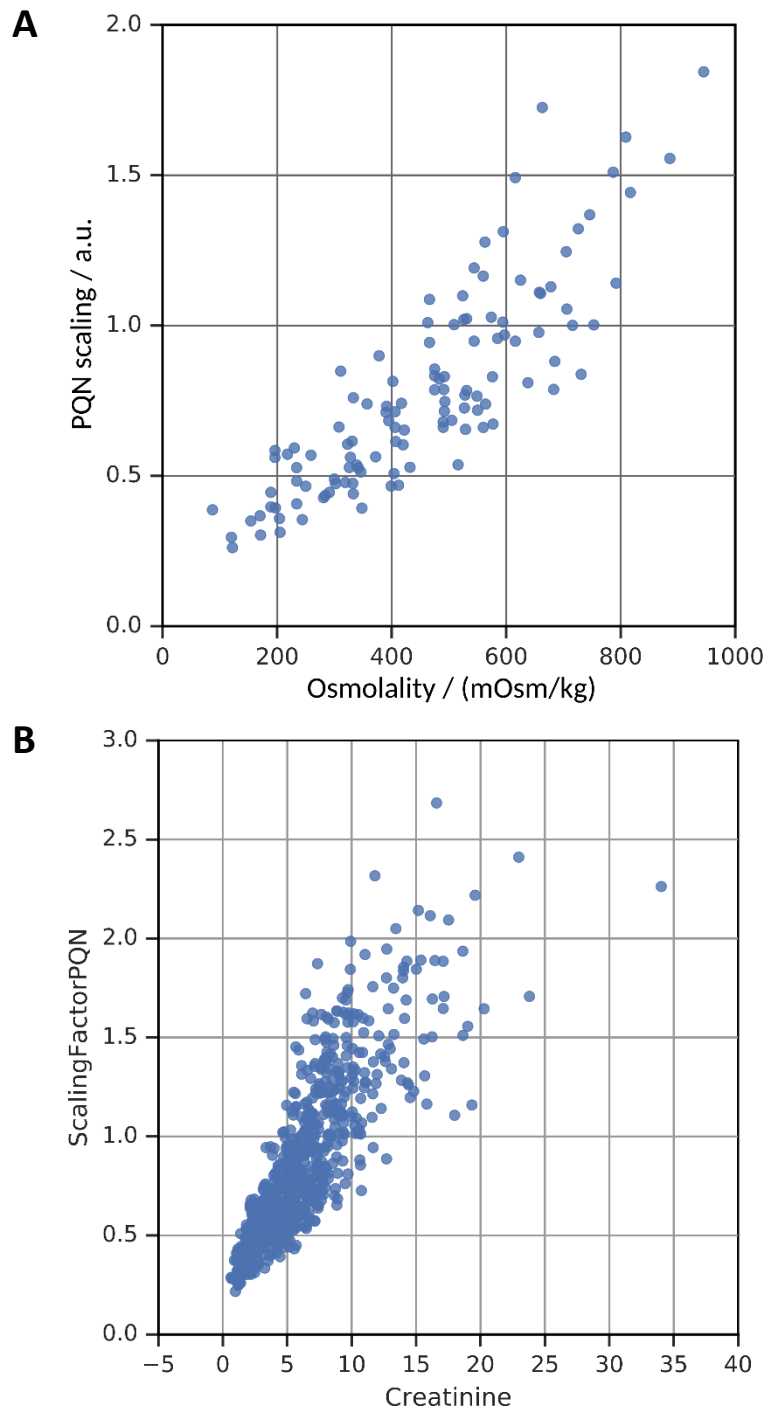

Supplement: S1 Fig — Correlation between probabilistic quotient normalization (PQN) and urinary osmolality (A) and urinary creatinine (B). To compensate for urine dilution differences, the metabolic data were normalized using PQN. This is a well-established normalization routine specifically developed for complex nuclear magnetic resonance spectroscopy (NMR), which considers the concentrations of all metabolites. The PQN scaling factor was correlated with (clinical laboratory-derived) urinary osmolality (A, r = 0.86). A similar correlation was found between PQN and urinary creatinine (B, r = 0.84). (PDF) [file pone.0233213.s001.pdf]
