# Supplementary material for: Urinary metabolites associate with the rate of kidney function decline in patients with autosomal dominant polycystic kidney disease
Source: PLoS One. 2020 May 22;15(5):e0233213. doi: 10.1371/journal.pone.0233213 (PMC7244119; doi:10.1371/journal.pone.0233213)
Supplement: S2 Fig — The plot showed no trend associated with fasting status. It was built using data matrix of the 29 quantified metabolites, and the model required seven components to cover the first 50% of the variance with 20% covered by the first two components. (PDF) [file pone.0233213.s002.pdf]

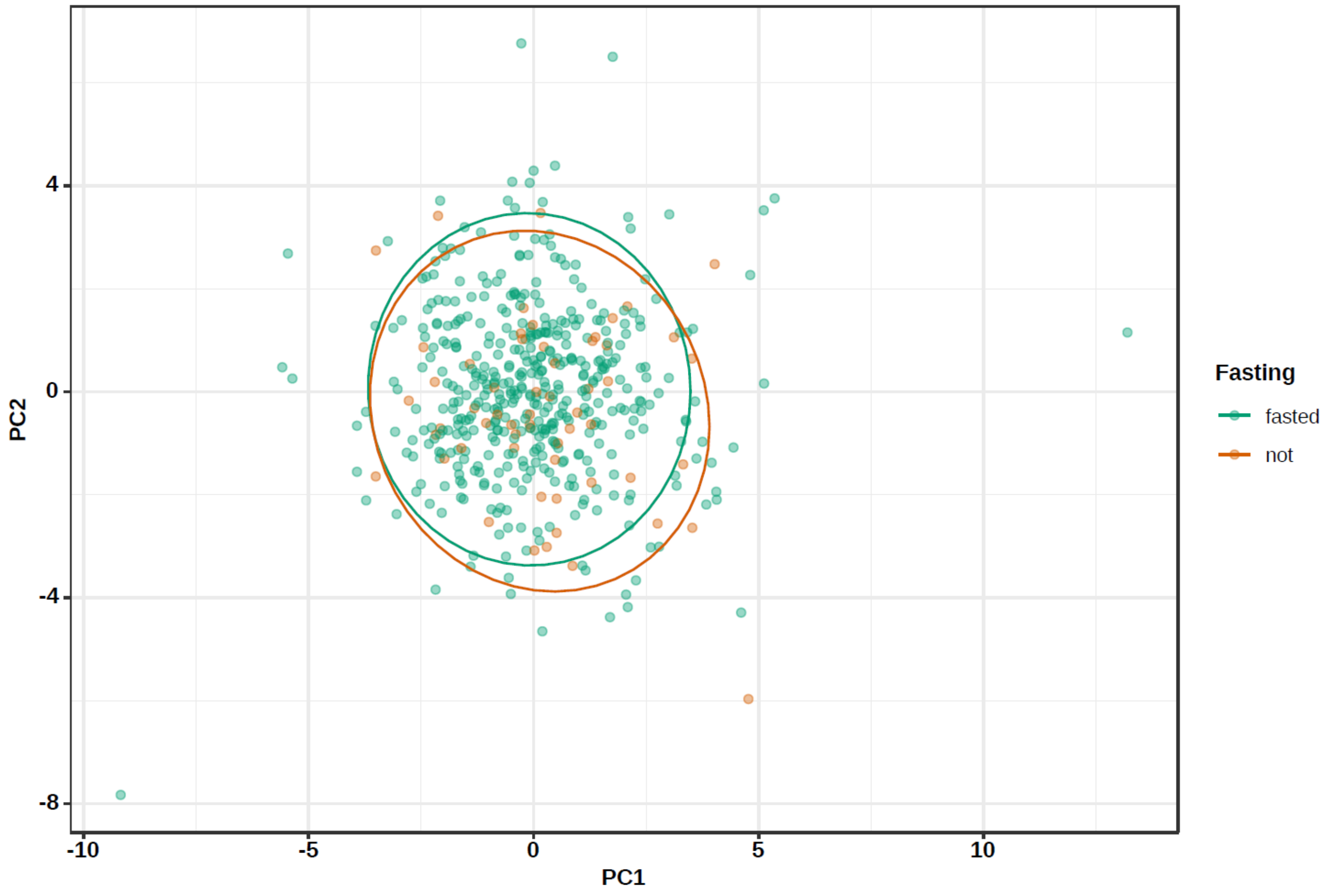

S2 Fig. A principal component analysis (PCA) score plot comparing the urinary metabolic profiles between fasting and non-fasting obtained urine samples. The plot showed no trend associated with fasting status. It was built using data matrix of the 29 quantified metabolites, and the model required seven components to cover the first 50% of the variance with 20% covered by the first two components.
