# Supplementary material for: Urinary metabolites associate with the rate of kidney function decline in patients with autosomal dominant polycystic kidney disease
Source: PLoS One. 2020 May 22;15(5):e0233213. doi: 10.1371/journal.pone.0233213 (PMC7244119; doi:10.1371/journal.pone.0233213)
Supplement: S3 Fig — Several urinary metabolites correlated with the actual eGFR. Significant Pearson correlations (r, p<0.05) are indicated by circles, blue and red indicating positive and negative correlations, respectively. The size of the correlation is indicated by the shade of the circles, and is defined by the colour bar. Correlation clusters and the correlations of eGFR with the metabolites are highlighted by black and red borders, respectively. (PDF) [file pone.0233213.s003.pdf]

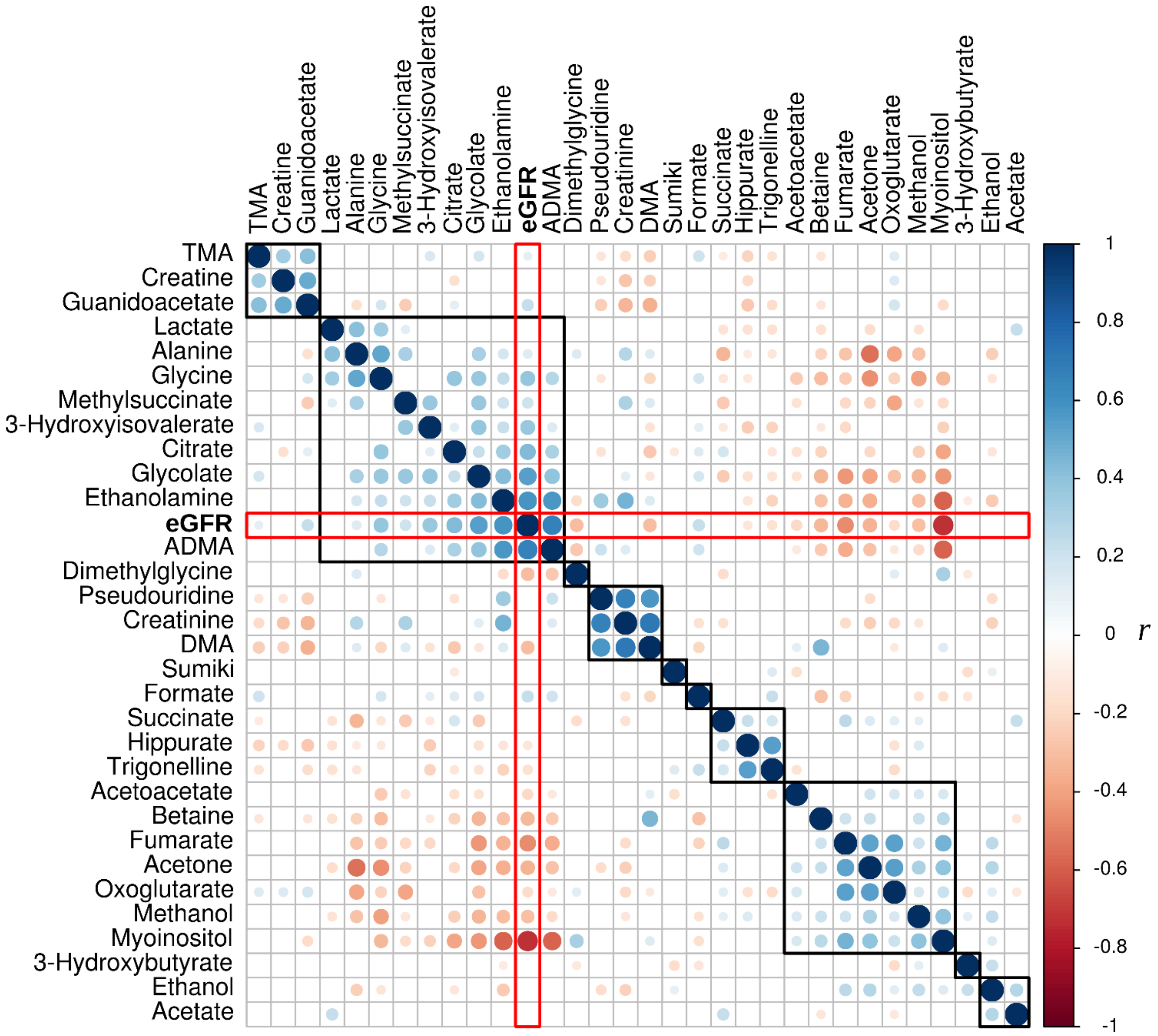

S3 Fig. Correlation map of all quantified metabolites built on the discovery cohort. Several urinary metabolites correlated with the actual eGFR. Significant Pearson correlations (*r*, $p<0.05$) are indicated by circles, blue and red indicating positive and negative correlations, respectively. The size of the correlation is indicated by the shade of the circles, and is defined by the colour bar. Correlation clusters and the correlations of eGFR with the metabolites are highlighted by black and red borders, respectively.
