# Supplementary material for: Urinary metabolites associate with the rate of kidney function decline in patients with autosomal dominant polycystic kidney disease
Source: PLoS One. 2020 May 22;15(5):e0233213. doi: 10.1371/journal.pone.0233213 (PMC7244119; doi:10.1371/journal.pone.0233213)
Supplement: S4 Fig — We performed model derivation based on a restricted cohort (n = 240) including patients with a follow-up of at least two years and/or four estimated GFR (eGFR) measurements to identify metabolites association with annual change in eGFR. The alanine/citrate ratio is most strongly associated with annual change in eGFR. Legend: x-axis; percentage increase in mean squared error (%IncMSE) when a variable is dropped, y-axis: metabolite ratios. (PDF) [file pone.0233213.s004.pdf]

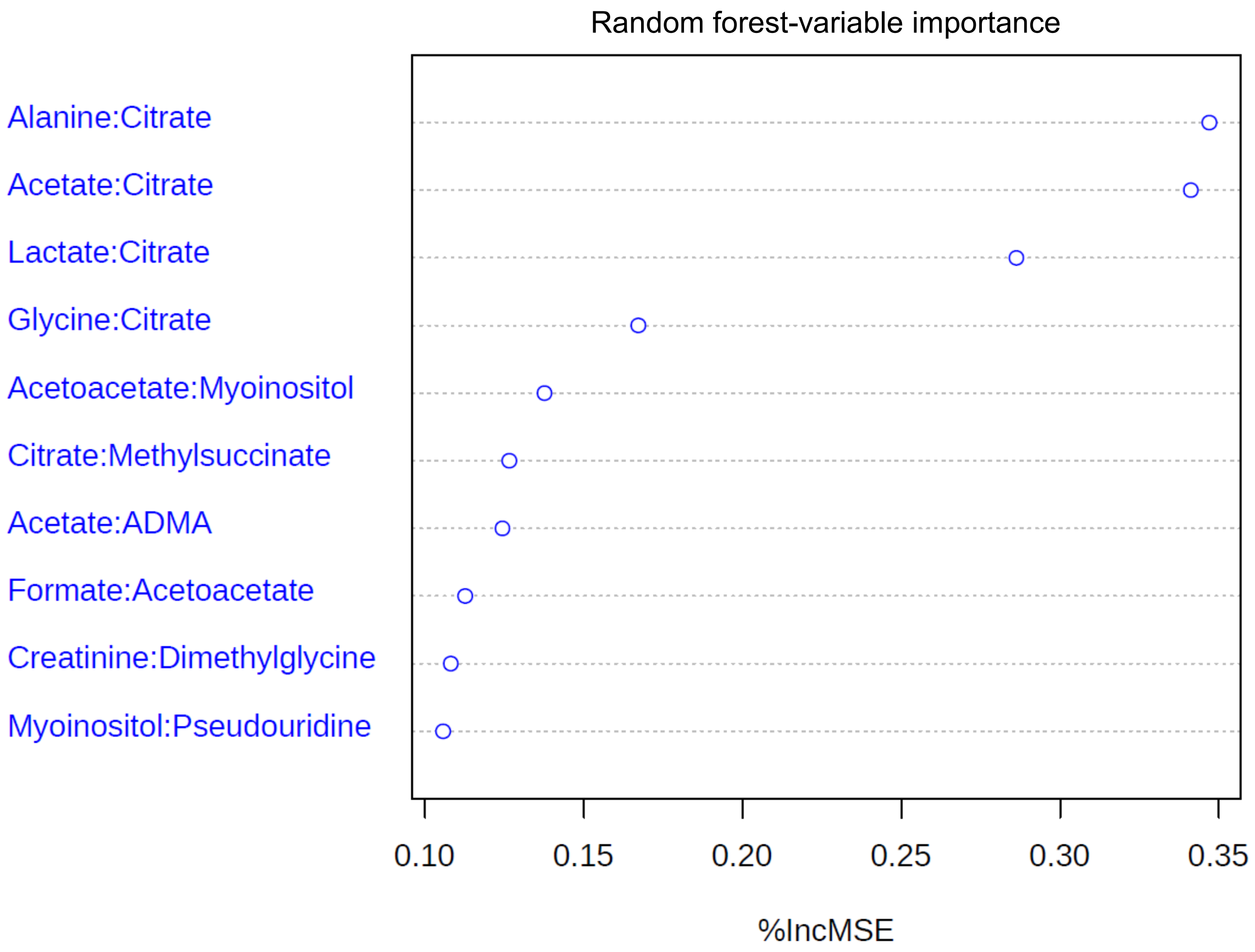

S4 Fig. Variable Importance Plot of a random forest regression model showing the top 10 of candidate predictors for estimated GFR (eGFR) progression. We performed model derivation based on a restricted cohort (n=240) including patients with a follow-up of at least two years and/or four estimated GFR (eGFR) measurements to identify metabolites association with annual change in eGFR. The alanine/citrate ratio is most strongly associated with annual change in eGFR. Legend: x-axis; percentage increase in mean squared error (%IncMSE) when a variable is dropped, y-axis: metabolite ratios.
