# Supplementary material for: Urinary metabolites associate with the rate of kidney function decline in patients with autosomal dominant polycystic kidney disease
Source: PLoS One. 2020 May 22;15(5):e0233213. doi: 10.1371/journal.pone.0233213 (PMC7244119; doi:10.1371/journal.pone.0233213)
Supplement: S5 Fig — The model (transformed to log2 eGFR) including four urinary metabolites (myo-inositol, asymmetric dimethylarginine (ADMA), 3-hydroxyisovalerate, creatinine) was associated with the actual eGFR in ADPKD (n = 338, blue dots) and non-ADPKD (n = 42, CKD stage 1–2; orange dots) patients (between the cohorts; r = 0.289, P = 0.027). (PDF) [file pone.0233213.s005.pdf]

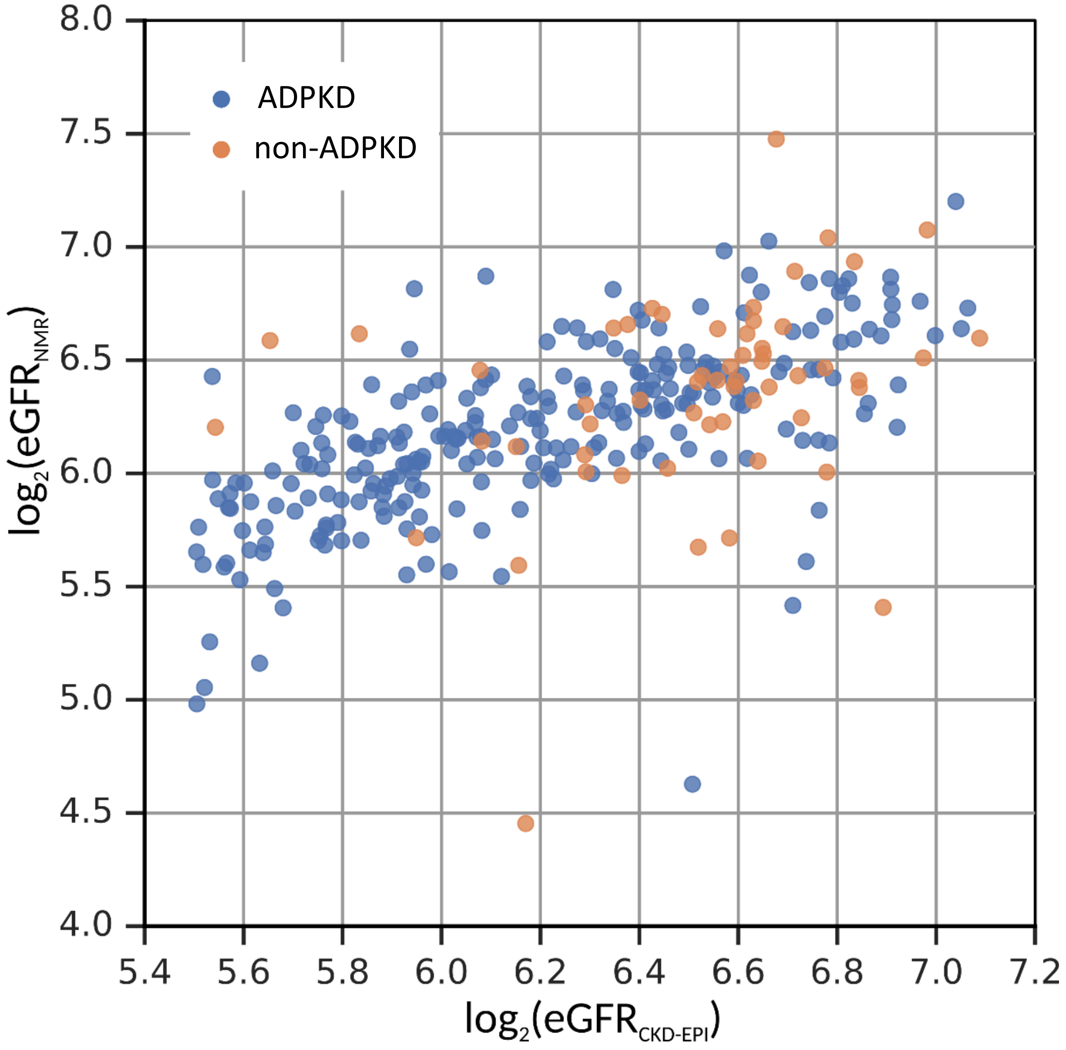

S5 Fig. Correlation between actual and predicted estimated glomerular filtration rate (eGFR) in patients with autosomal dominant polycystic kidney disease (ADPKD) and other chronic, renal disease patients (non-ADPKD). The model (transformed to log_2 eGFR) including four urinary metabolites (myo-inositol, asymmetric dimethylarginine (ADMA), 3-hydroxyisovalerate, creatinine) was associated with the actual eGFR in ADPKD (n=338, blue dots) and non-ADPKD (n=42, CKD stage 1-2; orange dots) patients (between the cohorts; r=0.289, P=0.027).
