# Supplementary material for: Urinary metabolites associate with the rate of kidney function decline in patients with autosomal dominant polycystic kidney disease
Source: PLoS One. 2020 May 22;15(5):e0233213. doi: 10.1371/journal.pone.0233213 (PMC7244119; doi:10.1371/journal.pone.0233213)
Supplement: S1 Table — Data in mean and SD. ADMA, asymmetric dimethylarginine; CKD, chronic kidney disease; DMA, dimethylamine; TMA, trimethylamine. *Between stages of CKD (Kruskal-Wallis test). (PDF) [file pone.0233213.s006.pdf]

S1 Table. PQN-corrected levels of the quantified urinary metabolites (n=29) stratified by CKD stage

Metabolites (mM)	Stage of CKD				*P* *
	1	2	3	4-5	
Creatinine	16.190 (4.884)	16.073 (4.464)	16.556 (4.403)	16.614 (4.104)	0.672
Alanine	0.376 (0.205)	0.281 (0.124)	0.266 (0.118)	0.312 (0.127)	9.07×10^{-08}
Betaine	0.921 (1.363)	1.021 (1.448)	1.250 (1.603)	1.489 (1.819)	2.94×10^{-07}
Creatine	0.718 (0.907)	0.501 (0.577)	0.434 (0.422)	0.378 (0.114)	0.309
Formate	0.285 (0.199)	0.233 (0.094)	0.200 (0.063)	0.181 (0.046)	4.26×10^{-06}
Lactate	0.428 (0.214)	0.361 (0.256)	0.365 (0.243)	0.447 (0.305)	0.001
Methanol	0.235 (0.126)	0.294 (0.145)	0.391 (0.256)	0.382 (0.238)	1.73×10^{-09}
Hippurate	4.720 (3.545)	4.497 (2.997)	5.186 (3.905)	5.405 (3.006)	0.216
Glycine	1.986 (1.381)	1.280 (0.754)	1.008 (0.587)	0.944 (0.488)	2.66×10^{-17}
DMA	0.469 (0.130)	0.470 (0.126)	0.531 (0.175)	0.574 (0.164)	4.91×10^{-06}
Acetate	0.145 (0.287)	0.070 (0.093)	0.066 (0.085)	0.113 (0.251)	0.002
Citrate	2.831 (1.320)	2.495 (1.312)	1.743 (1.014)	1.214 (0.756)	3.63×10^{-18}
Trigonelline	0.557 (0.533)	0.570 (0.483)	0.594 (0.508)	0.768 (0.498)	0.088
TMA	0.025 (0.008)	0.026 (0.008)	0.026 (0.008)	0.022 (0.008)	0.010
Succinate	0.161 (0.072)	0.142 (0.038)	0.140 (0.044)	0.145 (0.028)	0.277
Dimethylglycine	0.101 (0.035)	0.189 (0.986)	0.118 (0.039)	0.144 (0.037)	6.34×10^{-10}
Methylsuccinate	0.029 (0.049)	0.021 (0.010)	0.021 (0.012)	0.016 (0.006)	0.007
3-Hydroxyisovalerate	0.086 (0.026)	0.077 (0.026)	0.069 (0.019)	0.058 (0.018)	2.09×10^{-11}
Guanidoacetate	0.595 (0.303)	0.429 (0.257)	0.249 (0.293)	0.370 (0.148)	6.93×10^{-17}
Ethanolamine	0.748 (0.256)	0.673 (0.183)	0.588 (0.130)	0.384 (0.117)	4.67×10^{-19}
Acetone	0.024 (0.013)	0.042 (0.105)	0.037 (0.018)	0.040 (0.012)	3.41×10^{-13}
3-Hydroxybutyrate	0.277 (0.447)	0.274 (0.903)	0.229 (0.233)	0.214 (0.223)	0.151
Acetoacetate	0.061 (0.037)	0.077 (0.063)	0.062 (0.041)	0.074 (0.029)	0.001
Sumiki	0.046 (0.057)	0.038 (0.044)	0.038 (0.032)	0.037 (0.033)	0.425
Myoinositol	0.251 (0.353)	0.259 (0.103)	0.415 (0.255)	0.982 (0.521)	2.41×10^{-36}
Glycolate	0.545 (0.301)	0.430 (0.203)	0.356 (0.185)	0.198 (0.060)	2.47×10^{-20}
ADMA	0.033 (0.013)	0.027 (0.010)	0.020 (0.009)	0.011 (0.006)	7.01×10^{-33}
Oxoglutarate	0.367 (0.120)	0.440 (0.146)	0.473 (0.160)	0.441 (0.105)	8.81×10^{-07}
Pseudouridine	0.415 (0.107)	0.417 (0.101)	0.471 (0.121)	0.413 (0.106)	9.55×10^{-07}

Data in mean and SD. ADMA, asymmetric dimethylarginine; CKD, chronic kidney disease; DMA, dimethylamine; TMA, trimethylamine; PQN, probabilistic quotient normalization. *Between stages of CKD (Kruskal-Wallis test).
