# Supplementary material for: Urinary metabolites associate with the rate of kidney function decline in patients with autosomal dominant polycystic kidney disease
Source: PLoS One. 2020 May 22;15(5):e0233213. doi: 10.1371/journal.pone.0233213 (PMC7244119; doi:10.1371/journal.pone.0233213)
Supplement: S2 Table — ADPKD, autosomal dominant polycystic kidney disease; eGFR, estimated GFR; st β, standardized β. St β, F and P values were calculated using multivariable linear regression. (PDF) [file pone.0233213.s007.pdf]

S2 Table. Association between the urinary alanine/citrate ratio and annual change in eGFR in a randomly selected ADPKD cohort (n=350).

**Model**	**St. β**	**F**	***P***	**R^2**
Alanine/citrate ratio	-0.3765	50.08	1.03×10^{-11}	0.142

ADPKD, autosomal dominant polycystic kidney disease; eGFR, estimated GFR; st β, standardized β. St β, F and P values were calculated using multivariable linear regression.
